# Evaluation of the Growth Performance and Meat Quality of Different F1 Crosses of *Tengchong Snow* and *Xichou Black Bone* Chicken Breeds

**DOI:** 10.3390/ani14213099

**Published:** 2024-10-27

**Authors:** Zijian Li, Maida Mushtaq, Muhammad Khan, Jing Fu, Abdur Rahman, Yingxiang Long, Yong Liu, Xiannian Zi, Dawei Sun, Changrong Ge, Kun Wang

**Affiliations:** 1 College of Animal Science and Technology, Yunnan Agricultural University, Kunming 650201, China; lzj01071217@163.com (Z.L.); lyx2377494139@outlook.com (Y.L.); zxnzyx1119@163.com (X.Z.); gcrzal@126.com (C.G.); 2 Yunnan Animal Science and Veterinary Institute, Jindian, Panlong District, Kunming 650201, China; maida.ch17@gmail.com (M.M.); khanbwn011@gmail.com (M.K.); fj11260027@163.com (J.F.); sdw17787273236@163.com (D.S.); 3 Department of Animal Sciences, University of Veterinary and Animal Sciences, Jhang Campus, Jhang 35091, Pakistan; abdurrehman@uvas.edu.pk; 4 College of Animal Science and Technology, Yunnan Open University, Kunming 650101, China; lydzq05091025@163.com

**Keywords:** crossbreeding, growth performance, meat quality, Tengchong Snow chicken

## Abstract

Unlike other chicken breeds, Xichuan Black Bone (XBB) chickens are an established breed in China with excellent production performance and unique characteristics, including black meat, beaks, skin, bones, and legs, and they produce blue-shelled eggs. The Tengchong Snow (TS) chicken breed has relatively lower growth performance than commercial breeds but is considered one of the main genetic treasures of black meat in China. To improve the production and meat quality traits of the TS chickens by hybridization, the current study aimed to investigate the growth performance, carcass indices, meat quality physical properties, and muscle fiber traits of fiber traits of F1 crosses of TS with XBB chickens. Three groups of crossbreeding combinations were produced: (1) XT group (XBB  × TS ), (2) TX group (TS  × XBB ), and (3) TT group (TS  × TS ), with the TT group used as a control. A total of 725 healthy chicks (XT group: 247, TX group: 180, TT group: 298) were reared up to 20 weeks of age to estimate the growth performance and associated meat parameters. The results showed that the XT and TX groups had higher body weight and body size compared with the TT group (*p* < 0.05). Similarly, breast width, breast length, width of body, and carcass weights were also greater (*p* < 0.05) in the XT and TX groups compared with the TT group. Meat physical properties, including color, water-holding capacity, and tenderness, were improved (*p* < 0.05) for the XT and TX group compared to the TT group. The XT group had the better color of the leg muscles with the unique orientation of muscle fibers. Based on the results, the XT group is more in line with the future breeding direction as they have greater body weight, larger size, and lower abdominal fat. This study is a baseline technical reference for the protection, evaluation, and utilization of germplasm resources of Tengchong Snow chicken for screening the best matching lines and combinations with local chickens.

## Simple Summary

Tengchong Snow (TS) chickens have relatively lower growth performance than commercial breeds while considered a genetic treasure for their black meat. This study investigated growth performance, carcass attributes, meat physical properties, and muscle fiber traits of F1 crosses of TS with Xichuan Black Bone (XBB) chickens. Three F1 groups XT (XBB 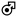 × TS 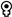), TX (TS 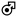 × XBB 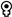), and TT (TS 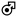 × TS 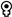, control) were raised to 20 weeks of age. Results showed that the XT and TX groups had higher body weight, larger body size, and improved meat properties compared to the TT group. The XT group, in particular, exhibited better leg muscle color, unique muscle fibers, and lower abdominal fat, making it a promising line for future breeding. This study provides a technical reference for utilizing and protecting the Tengchong Snow germplasm.

## 1. Introduction

In recent years, local chickens have become the first choice of consumers in many Asian countries including China, and they provide local farmers with daily essential meat and egg products and play an important supporting role in regional economic income. Compared with commercial broilers, native chicken breeds of China have better chewiness and richer nutrient content [1,2], creating a new market with great potential. Many indigenous chickens with unique genetic resources have been born in long-term natural selection or research-based artificial breeding. For example, the Dawei mountain miniature chicken is characterized by its small size and high metabolic capacity [3]. The Xishuangbanna fighting chicken is characterized by its thick legs and highly aggressive behavior [4], and the osseous chickens, such as Tengchong Snow chicken (TS) and Xichou Wu bone chicken, are characterized by the presence of abnormal melanin deposition in the muscles and internal organs [5]. These native chickens, especially the Black Bone chicken, are popular in China and its neighboring regions. Recent studies have also emphasized the potential of ostrich meat as a natural antioxidant [6], as well as to promote the development of organisms [7]. Therefore, the crossing of indigenous chickens with commercially established breeds can improve growth performance with a better feed conversion ratio that could result in the potential economic value of indigenous chickens with unique genetic resources.

Crossbreeding is a classical breeding method commonly used in poultry farming. Researchers and breeders often crossbreed to enhance specific traits by introducing qualities from a breed with specialized characteristics. For example, Tunim et al. [8] cross-bred commercial broilers with Thai indigenous chickens to improve fat deposition in Thai crossbred chickens. They found that the crossbreeding increased carcass fat content and expression of the peroxisome proliferator-activated receptor (PPAR) gene, which is related to fat deposition. This improvement was positively correlated with the degree of crossbreeding, showing that the more the chickens were crossbred, the higher the fat deposition. Another research by El-Tahawy et al. [9] revealed that the reproductive performance of a native Egyptian chicken named Sinai was improved by crossing it with a commercial breed, Lohmann Brown.

Crossbreeding experiments between two native chickens with different traits are also possible. In the recent past, Laenoi et al. [10] crossbred the two native Black Bone Thai breeds to improve their growth performance. They found superior growth performance of the hybrid progeny to that of the parents, especially in terms of body weight and feed conversion ratio. To date, fewer studies have been conducted on the use of crossbreeding to improve the growth performance of TS. In this study, Xichou Black Bone chicken (XBB) was crossbred with TS chicken. The XBB was originally developed in the mountainous Xichuan county of China, and it has five black features including a beak, skin, bones, legs, and meat. As demand grew, XBB became a key income source, leading to large-scale farming and the development of this unique breed. The TS is also a black meat-producing poultry germplasm; however, its growth performance is relatively lower than other commercial local breeds. The main objective of the current experiment was to improve the growth performance of the hydride of TS chicken by crossing with an established local breed named XBB. Moreover, it provides a theoretical reference for the efficient utilization of local chicken breed resources in Yunnan Province.

## 2. Materials and Methods

### 2.1. Experimental Animal and Feeding Management

The protocols and procedures of this experiment were approved by the Animal Use and Care Committee of the Yunnan Agriculture University (YAU), Kunming, China, before the research trial (Protocol ID: YAUACUC01). The XBB and TS chickens were reared in the experimental chicken farm of the YAU and were fed ad libitum, watered freely, routinely immunized, and standardized rearing management was implemented. The hybridization protocols used in this experiment were in accordance with Isa et al. [11]. Briefly, two selected parental lines were crossed in a reciprocal fashion using artificial insemination (AI) with a mating ratio of 1:5 to generate three populations: XT (XBB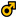 × TS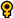), TX (TS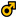 × XBB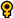), and TT (TS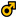 × TS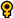, control). The TT group was used as a control in this study. The semen used for AI was fresh as well as undiluted for each cock and used at 2–day intervals. Egg collection was performed on the second day post-AI for 14 days and stored in the egg storeroom. The number of eggs set for incubation in a single hatch was 505, 536, and 400 for the XT, TX, and TT groups, respectively. A total of 725 healthy day-old chicks, 247, 180, and 298 for the XT, TX, and TT groups, respectively, were acquired, vaccinated, and transferred to the brooding pens. The rearing density was adjusted over time to 24–28 chicks/m^2^ by 4 weeks and 15–20 chicks/m^2^ from 4 to 7 weeks. The brooding conditions were standardized and identical across the groups. After 8 weeks, the chickens were transferred to triple cages until 20 weeks at 4–6 chicks/m^2^ density. The nutritional levels of the diets were maintained at the same level as the rearing and management conditions. The experimental chicken diets and chemical composition are shown in Table 1 and Table 2 respectively. The rearing conditions were homogenous across the groups and were in accordance with the established standards of the research poultry farm of the YAU. Briefly, the feed was offered twice (6:00 and 18:00 h) a day, 5% refusal was adjusted daily for ad libitum intake, fresh and clean water was ensured around the clock, and in-house air quality parameters (temperature fluctuated from 20.6 to 24.6 °C, whereas ammonia ranged from 9 to 24 ppm) were monitored regularly using the automatically installed system (Bestone Industrial Co., Ltd., Guangdong, China). The birds were immunized by following the established protocols of the YAU poultry research farm. The lighting program gradually reduced light exposure from 24 h at day-old to 10 h at 8 weeks old. The light was provided for 10 h every day for 12 weeks of growth. The lighting period was gradually raised by 1.5 h every week and achieved 16 h until 16 weeks of age. From 17 to 20 weeks, light was provided 17 h per day across the house.

### 2.2. Body Weight Measurements

To calculate the body weight, the individual birds of all three experimental flocks were weighed weekly from 0–140 days of age. In brief, the individual bird has a tag ring across the right leg with a specific identity number. After overnight fasting, every Sunday morning the birds’ weights were recorded using a digital weighing balance (capacity 10 kg ± 1 g; Xiamen Clarence Technology Co., Fuzhou, China).

### 2.3. Slaughtering Performance Measurements

At the age of 140 days, 50 cocks and 50 hens were selected from each group (a total of 300 from all groups), kept off-fed for 24 h, and body weight was recorded on an individual basis using a digital scale (capacity 10 kg ± 1 g; Xiamen Clarence Technology Co., Fuzhou, China). The body measurements were recorded of these birds using specific measurement methods referred to NY/T 823-2004 Nomenclature and Metric Statistical Methods of poultry production performance [12]. Briefly, breast width (distance between shoulder joints), breast depth (from the first thoracic vertebra to the keel), shank length (upper metatarsal joint to the middle of the third and fourth toes), keel length (front to the back of the keel), and pelvic width (distance between pelvic bones) were measured with a slide caliper (Delixi Electric, Wenzhou, China). Body slope length (shoulder to ipsilateral ischial tuberosity), shank length (hock to toe), and shank girth (shin’s center circumference) were measured using a soft tape measure (Deli, Ningbo, China). Then, the slaughtering of all birds was performed by using neck bleeding and wet plucking methods. The slaughtering performance and sampling were carried out by following the procedures and protocols of Huo et al. [13]. The de-feathered carcass, including the head and feet, was weighed. The trachea, esophagus, gastrointestinal system, crop, spleen, pancreas, gallbladder, and gonads were manually removed to determine the half-eviscerated weight. The head, feet, heart, liver, gizzard, glandular stomach, and abdominal fat were then removed, and the eviscerated weight was recorded. The head, abdominal fat, claws, wings, breast, and thighs were also weighed. Dressing percentage was calculated as the ratio of cold carcass weight to body weight. The pectoral major (incised lateral medial position to keel bone of right side of the breast) and hamstring muscles (from lateral position of the right thigh) were sampled (0.5 × 0.5 × 1.0 cm^3^), immediately dipped into liquid nitrogen, and then stored at −80 °C for myofiber determination. The skin, visible fat, and excess connective tissue were removed from the left pectoral and hamstring muscles, sampled, vacuum packed, and stored at 4 °C for meat analysis.

### 2.4. Meat Quality Physical Properties Analysis

The physical properties associated with meat quality were determined by following the methods of Xue et al. [14] and Weng et al. [15]. The pH of the pectoral and hamstring muscles was recorded at 45 min and 24 h after slaughter by using a portable pH meter (Model E-201-C, Shanghai Leimagnet Instrument Factory, Shanghai, China). At 45 min after slaughtering, trimmed pieces of each of the pectoral and hamstring muscles were analyzed by using a portable colorimeter (Model Minolta-CR200, Shenzhen Xinyuan Electronic Instrument Co., Ltd., Guangdong, China) to estimate the color coordinates (L* = brightness, a* = redness, and b* = yellowness) of the meat. Moreover, the pectoral and leg muscles of the same parts were weighed, and the rate of water loss by pressing was measured by a strain-type unconfined pressure meter (Model WW-2A, Nanjing Soil Instrument Factory Co., Ltd., Nanjing, China). The initial weight of the muscle (P1) and the weight after pressing (P2) were recorded, and the pressure losses were calculated using Equation (1). The meat samples were trimmed (2 cm × 2 cm × 1 cm) and the weight was recorded as C1 and then wrapped into a plastic pocket equipped with a thermometer and placed in water at 80 °C until the temperature in the pocket rose to 74 °C. The weight of the cooked meat was recorded as C2 and the cooking losses were calculated using Equation (2).

Pressing losses (%) = [(P1 − P2)/P1] × 100(1)

Cooking losses (%) = [(C1 − C2)/C1] × 100(2)

Cooked meat samples after determining cooking losses were subjected to a tenderizer (Model C-LM25, Beijing Tianxiang Feiyuwei Instrument Co., Ltd., Beijing, China) and shear force was applied three times for each sample to determine the meat tenderness.

### 2.5. Histological Characterization of Muscle Fibers

In this experiment, muscle samples were subjected to a frozen sectioning technique to prepare the muscle sections. Briefly, muscle samples were placed in an embedding box (25 mm × 40 mm × 5 mm) partially filled with OCT and covered completely by adding more OCT. The embedding boxes containing the samples were snap-frozen in liquid nitrogen. Cross-sectional sections (10 μm thick) of the muscle fibers were prepared using a cryostat at −25 °C (Leica, Wetzlar, Germany), transferred to adhesive glass slides, air-dried, and stored at −20 °C for subsequent histological staining.

Muscle fiber morphological features were stained with hematoxylin and eosin by following the established protocols: hematoxylin, 5 min; water wash, 10 min; 1% acidic alcohol differentiation solution, 25 s; water wash, 15 min; eosin, 3 min; water wash, 2 min. After staining, sections were dehydrated and cleared before sealing with neutral resin. The diameter, single cross-sectional area, and density of muscle fibers were examined at 200× magnification using a Nikon ECLIPSE TI-S image acquisition system (Nikon Corporation, Tokyo, Japan). Muscle fiber diameter and cross-sectional area were calculated by image analysis using the image analysis system Image Pro-plus 5.02. For each sample, three images were taken to count 100 myofibers with clear and intact morphology, respectively. Measurements were made on samples of 36 chickens (half of the cocks and half of the hens) from each of the three groups.

Muscle fiber type was determined using myosin ATPase activity, following the method. The procedure involved a pre-incubation step at pH 4.3 for 10 min at 37 °C, followed by three distilled water washes (2 min each). ATPase incubation was carried out at pH 9.6 for 45 min at 37 °C, followed by incubation in 10% calcium chloride solution for 3 min, and then in 2% cobalt nitrate solution for 3 min at room temperature (22 °C). After three additional distilled water washes (2 min each), sections were incubated in 1% ammonium sulfide solution for 3 min, followed by a final distilled water wash for 5 min. Muscle fibers were then classified based on staining intensity: oxidized fibers (appearing dark) and glycolytic fibers (appearing light). Intact muscle bundles were selected, and the oxidized and glycolytic fibers were counted separately.

### 2.6. Statistical Analysis

The raw data was normalized using QQ plots (SPPS, Chicago, 18.0 version). The data was analyzed by One-way analysis of variance in SPPS software. The means were compared using the LSD test. Results were expressed as mean ± standard deviation (SD), with a significance level set at *p* < 0.05 to indicate statistically significant differences.

## 3. Results

### 3.1. Growth Performance and Carcass Attributes

**Figure 1:**
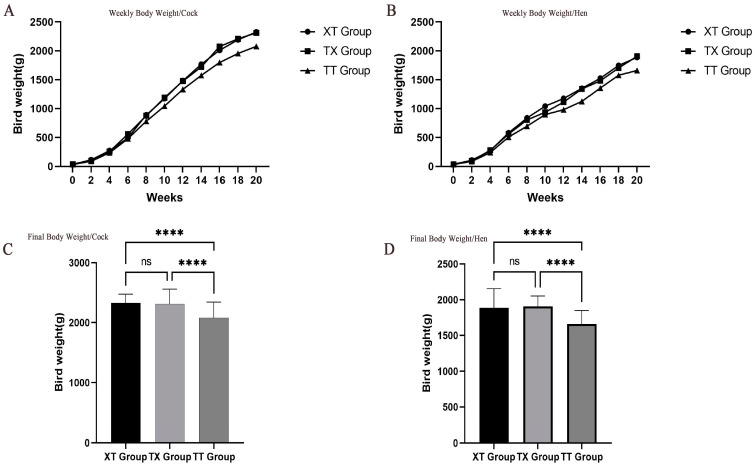
Weekly body weight (**A**,**B**) and final body weight (**C**,**D**) of cocks (**A**,**C**) and hens (**B**,**D**) of different crosses of Tengchong Snow and Xichou Black Bone chicken breeds. **** donated to significant difference (*p* < 0.05) across different groups.

The changes in body weights of the three groups of chickens from birth to week 20 are shown in Figure 1, which shows that the body weights of the XT and TX groups were significantly higher than those of the TT group (*p* < 0.05). However, body weight changes between the XT and TX groups were non-significant (*p* > 0.05). The results of growth performance and carcass indexes of the cocks and hens of the three groups are given in Table 3. The body measurement values including slope length, breast width, breast depth, pelvis width, shank length, and shank girth were greater (*p* < 0.05) in the XT and TX cocks compared to the TT cocks. Similarly, body slope length, breast width, breast depth, and pelvis width of the TX and XT hens were greater (*p* < 0.05) compared with the TT hens. The carcass performance, including body weight, carcass weight, half-eviscerated weight, dressing percentage, and half-eviscerated yield of the XT and TX cocks were greater (*p* < 0.05) than that of the TT cock. However, the keel bone length, abdominal fat weight, and head weight of the TT cocks were greater (*p* < 0.05) than those of the XT and TX cocks. The body weight, carcass weight, half-eviscerated weight, and dressing percentage of the XT and TX hens were greater (*p* < 0.05) than those of the TT hens. However, head weight, claws weight, and leg muscle rate were greater (*p* < 0.05) for the TT hens across the groups.

### 3.2. Comparison of Muscle Fiber Properties with Physical Properties

**Figure 2:**
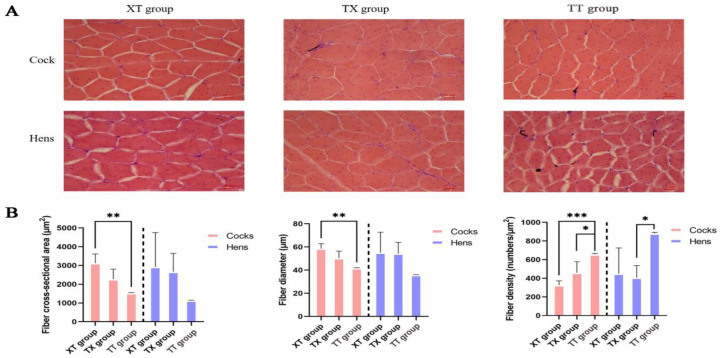
Morphological characteristics of thoracic muscle fibers in chickens. (**A**) Hematoxylin and eosin staining. 200× magnification was used. (**B**) Comparison of pectoral muscle myofiber characteristics. *, **, and*** donated to significant difference (*p* < 0.05) across different groups.

**Figure 3:**
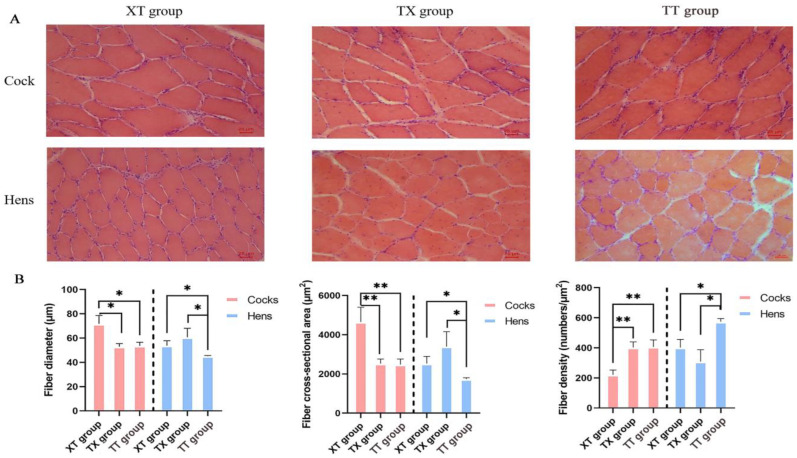
Morphological characteristics of thigh muscle fibers in chickens. (**A**) Hematoxylin and eosin staining. A 200× magnification was used. (**B**) Comparison of thigh muscle fiber characteristics. **, and * donated to significant difference (*p* < 0.05) across different groups.

**Figure 4:**
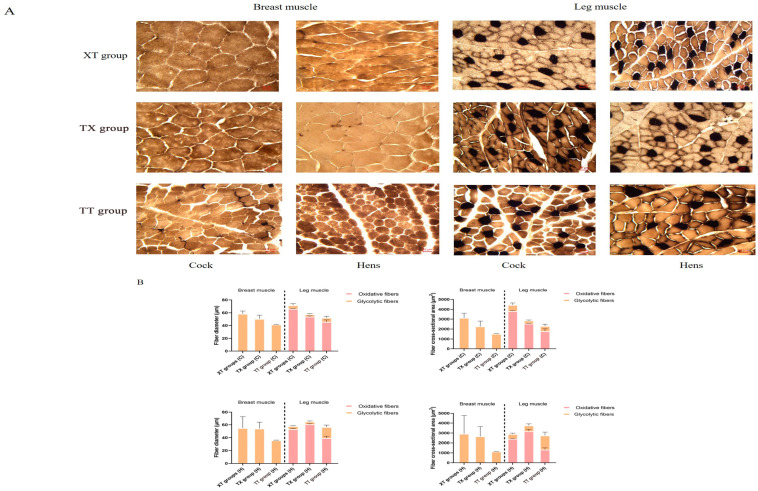
Composition of muscle fiber types in chicken muscles. (**A**) Analysis of ATPase staining of two muscle tissues. Glycolytic fibers are light in color; dark fibers are oxidized fibers. (**B**) Myofiber diameters and cross-sectional areas of glycolytic and oxidative fibers in two muscle tissues of XT and TX groups.

**Figure 5:**
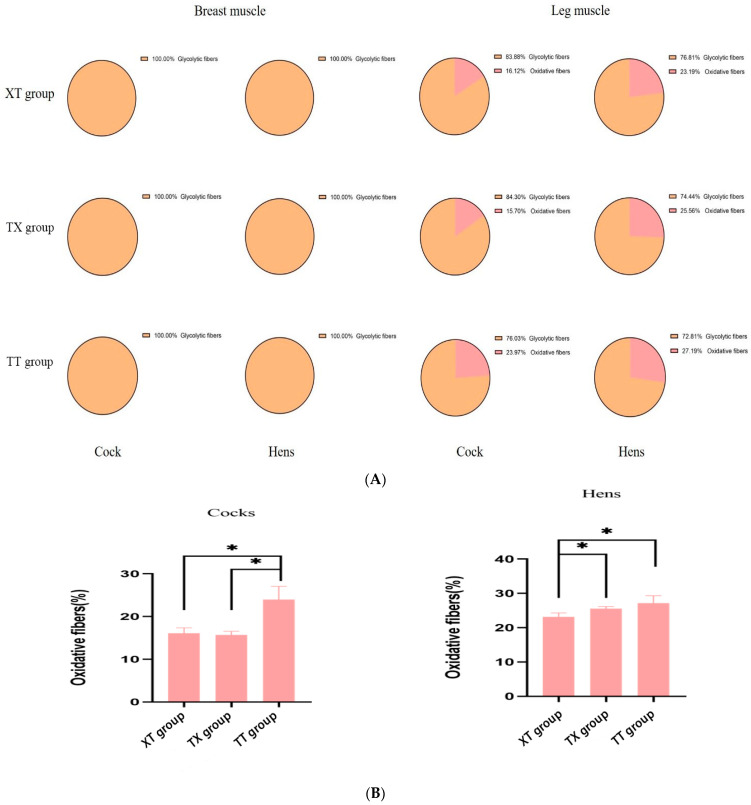
Comparison of myofiber types in two muscle tissues of chickens. (**A**) Percentage of glycolytic and oxidative fibers in chest or leg muscles. (**B**) Comparison of the percentage of oxidized fibers in the leg muscles of the three groups. * donated to significant difference (*p* < 0.05) across different groups.

The physical properties of the meat are shown in Table 4. In the cock group, the 45 min pH of leg muscle in the XT group was greater (*p* < 0.05) than TX and TT groups. The pH values recorded after 24 h of the leg muscle were greater (*p* < 0.05) for the XT and TX cocks as compared to the TT cocks. The color L* and b* values for leg and breast meat were also greater (*p* < 0.05) for the XT and TX cocks, whereas, pressing losses were lower (*p* < 0.05) for the TX and XT cocks as compared to the TT cocks. The cooking losses of breast and leg meat were greater (*p* < 0.05) for the TT cocks compared to the rest of the groups. In the hen group, the pH value recorded after 24 h was greater (*p* < 0.05) of the leg muscle of the XT group than the TX and TT groups. Similarly, meat color coordinates such as L* and b* values were greater (*p* < 0.05) for the XT hens than the TT hens. However, pressing losses were lowered (*p* < 0.05) in the meat of the TX hens compared to the TT hens. The cooking losses were higher (*p* < 0.05) for the meat of the TT hens as compared to the rest of the groups. The share force (tenderness) values were lower (*p* < 0.05) for the TT hens than the TX and TT groups. In addition, recent studies have shown that meat quality is closely related to muscle fiber traits [16], so we used histological staining to analyze the muscle tissues of chickens in the three groups. As shown in Figure 2 and Figure 3, compared to the three groups of the cocks, the XT group had the largest diameter and cross-sectional area of pectoral and hamstring muscle fibers, but the number of muscle fibers in a 1 mm^2^ area was lower (*p* < 0.05). Compared to the three groups of hens, the XT and TX groups had a greater (*p* < 0.05) diameter and cross-sectional area of leg muscle fibers than the TT group, and although there was no significant difference in pectoral muscle fiber characteristics between the XT and TX groups. The TX group had a significantly greater (*p* < 0.05) diameter and cross-sectional area of leg muscle fibers than the XT group and a smaller number of muscle fibers in a 1 mm^2^ area of leg muscle fibers. We further assessed the type of myofiber composition in the pectoral and leg muscles of the three groups using ATPase staining (shown in Figure 4 and Figure 5). The pectoral muscle myofibers of all three groups of chickens consisted almost entirely of glycolytic fibers, while the leg muscle myofibers were mainly composed of oxidative and glycolytic fibers.

## 4. Discussion

Most chickens worldwide are sold based on body weight, and improving chick weight directly affects their final economic value [17]. In this experiment, although the body weight of F1 crosses of Tengchong Snow chickens at 140 days of age was improved, there was no significant difference between the two crossbreeding combinations. It is important to continue analyzing the carcass attributes, which is one of the most critical economic parameters for farm animals. The primary meat attributes such as meat pH, color, tenderness, and cooking losses have a direct influence on meat quality and consumer preferences. Many factors affect the carcass attributes, such as animal strain, age, nutrition, and crossbreeding combinations [18,19,20]. In this study, crossbreeding combinations were the main cause of differences in the carcass traits in the F1 generation population. The breast muscle development of crossbred progeny was more favorable when Xichou Black Bone chicken was used as the parent. Conversely, using Tengchong Snow as the parent directly affected leg muscle development in the offspring, especially in hens. Due to the different muscle development patterns in the offspring of the two crosses, it is insufficient to conclude which combination is superior. However, excessive fat deposition in broilers can reduce their economic value, as consumers generally prefer chicken meat with low-fat content [21]. The results of this experiment found that by crossing these two breeds, the abdominal fat in the XT group was decreased. Meat is the primary end product of poultry, and skeletal muscle is one of the main components of meat, directly affecting meat yield and quality. In this experiment, the meat quality and muscle fiber characteristics of crossbred progeny were used as evaluation criteria for the improvement effect. However, the results did not fully align with other studies on improving the meat quality of indigenous chickens through crossbreeding [22], possibly due to differences in the selection of crossbreeding parents. The study showed that pectoral muscle fiber diameter was positively correlated with body weight and pectoral muscle rate, meaning that individuals with greater body weight also had larger muscle fiber diameters [23], which is consistent with the present study. It is worth noting that the pectoral muscle rate was higher in the XT group than in the TX group, although there was no significant difference in muscle fiber diameter between the two groups. Therefore, it is hypothesized that body weight has a greater effect on pectoral muscle fiber diameter. So far, no correlation has been established between leg muscle fiber traits and the carcass attributes. However, in this study, leg muscle fiber diameter and cross-sectional area were also larger in the XT group, which had a larger body size. Two factors may contribute to this result. First, Lukasiewicz et al. found that leg muscle fibers in fast-growing chickens were smaller and only half the size of those in slow-growing chickens, suggesting that genotype can directly influence leg muscle fiber development [24]. Second, since the number of muscle fibers in chickens is fixed before birth, postnatal muscle fiber development is primarily hypertrophic [25]. This process is directly or indirectly regulated by various factors, including coding and non-coding RNAs [26]. These differences in leg muscle fiber development across the three groups of chickens need further verification. The leg muscles of Tengchong Snow chickens contain more oxidative fibers, making them redder and more tender. Studies have shown that a large amount of myoglobin is usually found in oxidative fibers [27], and the redox state of myoglobin in muscle can directly influence meat color [28]. Additionally, higher tenderness is typically associated with a higher proportion of oxidative fibers [29]. Comparing the two crossbred groups, the TX group had a higher percentage of oxidative fibers in the leg muscles, making them redder and darker than those of the XT group. The color coordinates, such as L* value, are donated to the brightness of the meat. In this experiment, the significant difference in the L* values for the muscles suggests varying amounts of melanin across different groups, which is considered to be one of the factors affecting meat color. Studies have shown that the presence of melanin contributes to changes in muscle L* value, with greater melanin presence leading to a lower L* value [30]. In this study, although there was no statistical difference in muscle L* between the two crossbreeding groups and Tengchong Snow chickens, the overall higher L* suggests that crossbreeding may affect melanin deposition to some extent.

## 5. Conclusions

Based on the results of the current study, crossing of the Tengchong Snow chicken with Xichou Black Bone chicken resulted in improved growth performance with better body conformation of the F1 crosses (XT and TX). Among the crosses, the XT group had greater body weight, larger body size, and lower abdominal fat. This study provides technical references for the conservation, evaluation, and utilization of the Tengchong Snow chicken, as well as data support for screening the best supporting line combinations of local chickens.

## Figures and Tables

**Table 1:** Feed formulation on a dry basis.

Components	1–7 Weeks (%)	8–20 Weeks (%)
Corn	63.26	67.19
Soybean meal	30.2	18.88
Wheat bran	0.00	10.00
Fishmeal	2.50	0.00
Coarse stone powder	0.40	0.46
Fine stone powder	0.71	0.60
Dicalcium phosphate	1.50	1.50
Methionine	0.08	0.07
Salt	0.35	0.30
^1^ Commercial premix	1.00	1.00

^1^ Commercial premix contained Fe, 39 g; Mn, 36 g; Cu, 7.2 g; Zn, 25 g; I, 1.15 g; Cr, 0.12 g; Se, 0.18 g; Vit A, 23 MIU; Vit D3, 4.6 MIU; Vit E, 68.0 g; Vit K, 7.0 g; Vit B1, 6.0 g; Vit B2, 21.0 g; Vit B6, 6.0 g; Vit B12, 0.02 g; Niacin, 64.0 g; Calcium-D-pantothenate, 29.0 g; Folic acid, 4.5 g; Biotin, 0.24 g; and Vit C, 96.0 g.

**Table 2:** Diet compositional characteristics on a dry basis.

Nutrient Contents	1–7 Weeks (%)	8–20 Weeks (%)
Dry matter (%)	89.90	90.10
Crude protein (%)	22.15	17.56
Metabolizable energy (Kcal/kg DM)	3112.89	3057.23
Crude fiber (%)	3.55	4.00
Ether extract (%)	2.17	2.01
Ash (%)	3.46	3.01
Calcium (%)	0.76	0.98
Phosphorous (%)	0.36	0.43

**Table 3:** Comparative growth performance and carcass attributes of different F1 crosses of Tengchong Snow and Xichou Black Bone chicken breeds.

Indices	XT Group		TX Group		TT Group	
	Cocks	Hens	Cocks	Hens	Cocks	Hens
Body slope length (mm)	197.38 ± 14.21 ^A^	182.38 ± 10.20 ^a^	192.15 ± 10.27 ^B^	177.23 ± 9.58 ^b^	194.64 ± 10.97	164.60 ± 10.30 ^c^
Breast width (mm)	82.52 ± 5.27 ^A^	71.36 ± 4.59 ^a^	76.81 ± 3.65 ^B^	72.51 ± 3.92 ^a^	71.87 ± 9.35 ^C^	66.50 ± 4.7 ^b^
Breast depth (mm)	90.13 ± 6.14 ^A^	80.41 ± 7.49 ^a^	87.62 ± 4.31 ^B^	79.20 ± 7.39 ^a^	82.15 ± 7.37 ^C^	72.23 ± 5.32 ^b^
Pelvis width (mm)	87.07 ± 4.73 ^A^	92.60 ± 5.74 ^a^	82.57 ± 3.71 ^B^	84.65 ± 4.90 ^b^	76.33 ± 9.51 ^C^	75.30 ± 5.90 ^c^
Keel bone length (mm)	118.28 ± 9.57 ^A^	102.86 ± 4.01	116.23 ± 7.82 ^A^	102.80 ± 2.78	123.05 ± 14.34 ^B^	102.10 ± 5.9
Shank length (mm)	100.52 ± 11.76	80.67 ± 5.01	102.28 ± 5.37 ^A^	81.91 ± 6.20 ^a^	98.49 ± 8.78 ^B^	79.10 ± 3.5 ^b^
Shank girth (mm)	4.43 ± 0.25 ^A^	3.79 ± 0.17 ^a^	4.70 ± 0.21 ^B^	3.91 ± 0.12 ^b^	4.41 ± 0.60 ^A^	3.64 ± 0.40 ^c^
Body weight (g)	2326.90 ± 148.66 ^A^	1888.8 ± 265.99 ^a^	2311.40 ± 250.17 ^A^	1907.8 ± 143.52 ^a^	2078.2 ± 263.40 ^B^	1660.8 ± 339.5 ^b^
Carcass weight (g)	2037.20 ± 187.43 ^A^	1614.40 ± 234.94 ^a^	2032.95 ± 220.23 ^A^	1687.40 ± 117.68 ^a^	1834.15 ± 237.56 ^B^	1419.95 ± 336.7 ^b^
Half eviscerated weight (g)	1900.20 ± 153.76 ^A^	1502.75 ± 267.88 ^a^	1911.15 ± 230.38 ^A^	1514.33 ± 117.48 ^a^	1761.46 ± 245.67 ^B^	1329.70 ± 302.83 ^b^
Eviscerated weight (g)	1639.10 ± 147.42 ^A^	1206.25 ± 204.80	1582.45 ± 210.06	1240.10 ± 109.52	1518.85 ± 197.12 ^B^	1211.81 ± 238.18
Breast weight (g)	228.25 ± 24.15	215.69 ± 91.40 ^a^	223.65 ± 33.83	201.85 ± 30.26	224.15 ± 38.38	181.89 ± 49.55 ^b^
Thighs weight (g)	425.18 ± 22.85 ^A^	224.05 ± 40.12 ^a^	398.94 ± 22.25 ^B^	268.00 ± 52.76 ^b^	422.35 ± 78.81 ^A^	235.65 ± 35.34 ^a^
Abdominal fat weight (g)	44.95 ± 32.68 ^A^	119.95 ± 74.35 ^a^	51.40 ± 25.63 ^A^	88.10 ± 29.82 ^b^	66.31 ± 15.74 ^B^	106.25 ± 52.11
Wings weight (g)	197.10 ± 21.92	138.80 ± 23.14	194.10 ± 32.96	135.35 ± 50.38	193.15 ± 35.67	138.9 ± 21.19
Head weight (g)	99.00 ± 12.99 ^A^	47.40 ± 5.50 ^a^	95.70 ± 21.72 ^A^	47.65 ± 4.80 ^a^	129.03 ± 32.97 ^B^	51.65 ± 8.16 ^b^
Claws weight (g)	84.80 ± 10.17 ^A^	51.45 ± 9.33 ^a^	101.85 ± 37.08 ^B^	55.75 ± 8.01 ^b^	111.3 ± 31.41 ^B^	58.75 ± 8.86 ^b^
Dressing (%)	91.08 ± 2.72 ^A^	90.72 ± 2.03 ^a^	89.80 ± 2.63 ^B^	89.63 ± 3.25 ^a^	88.26 ± 2.56 ^C^	85.44 ± 3.32 ^b^
Half-eviscerated yield (%)	85.42 ± 1.67 ^A^	79.87 ± 3.26	84.31 ± 1.47 ^B^	80.44 ± 4.19	81.70 ± 2.13 ^C^	79.83 ± 1.34
Eviscerated yield (%)	72.46 ± 3.02 ^A^	63.72 ± 3.62 ^a^	73.14 ± 3.78 ^A^	69.17 ± 3.29 ^b^	66.67 ± 3.16 ^B^	67.70 ± 2.14 ^c^
Breast muscle (%)	14.14 ± 1.54 ^A^	15.80 ± 1.77 ^a^	11.73 ± 1.35 ^B^	13.31 ± 1.52 ^b^	14.76 ± 1.27 ^C^	15.00 ± 2.11 ^c^
Leg muscle (%)	25.94 ± 4.51	18.03 ± 1.42 ^a^	25.21 ± 3.54	21.48 ± 2.72 ^b^	24.80 ± 4.32	23.34 ± 4.21 ^c^

The superscripts ^A–C^ and ^a–c^ were used to separate means within a row for significant differences (*p* < 0.05) in the cocks and hens groups, respectively. The means among rows without these superscripts indicate a non-significant difference between groups (*p* > 0.05).

**Table 4:** Comparative meat physical properties of different crosses of Tengchong Snow and Xichou Black Bone Chicken breeds.

Item			pH		Meat Color			Pressing Loss (%)	Cooking Loss (%)	Shear Force (N)
			45 min	24 h	L*	a*	b*			
XT Group	Cocks	Breast	6.13 ± 0.09	6.05 ± 0.21	41.01 ± 3.13 ^A^	7.00 ± 1.89 ^A^	8.73 ± 1.44 ^A^	21.07 ± 3.29 ^A^	14.46 ± 2.79 ^B^	3.65 ± 0.89 ^A^
		Leg	6.32 ± 0.11 ^A^	6.27 ± 0.14 ^A^	39.35 ± 2.32 ^A^	9.06 ± 1.02 ^A^	7.31 ± 1.45 ^A^	20.41 ± 4.19 ^A^	14.22 ± 1.09 ^A^	3.19 ± 0.66 ^A^
	Hens	Breast	6.12 ± 0.13 ^a^	6.19 ± 0.21	42.91 ± 2.79 ^a^	5.58 ± 1.67 ^a^	8.72 ± 2.19 ^a^	19.91 ± 3.24 ^a^	13.04 ± 2.11 ^a^	3.82 ± 1.1 ^a^
		Leg	6.34 ± 0.24 ^a^	6.35 ± 0.18 ^a^	41.95 ± 3.27 ^a^	3.28 ± 0.89 ^a^	7.59 ± 2.19 ^a^	21.56 ± 1.22 ^a^	14.85 ± 2.26 ^a^	3.12 ± 1.43 ^a^
TX Group	Cocks	Breast	6.10 ± 0.12	6.04 ± 0.17	40.39 ± 2.89 ^A^	6.88 ± 1.19 ^A^	7.77 ± 0.79 ^B^	22.08 ± 2.88 ^A^	15.54 ± 2.62 ^A^	4.37 ± 1.02 ^B^
		Leg	6.20 ± 0.19 ^B^	6.25 ± 0.09 ^A^	39.20 ± 2.01 ^A^	9.39 ± 1.14 ^A^	6.20 ± 1.18 ^B^	21.25 ± 3.21 ^A^	14.93 ± 0.89 ^A^	2.32 ± 0.73 ^B^
	Hens	Breast	6.20 ± 0.07 ^b^	6.21 ± 0.15	40.59 ± 3.01 ^b^	5.32 ± 1.89 ^a^	7.19 ± 1.85 ^b^	23.09 ± 4.32 ^b^	16.16 ± 3.69 ^b^	4.42 ± 1.23 ^b^
		Leg	6.16 ± 0.16 ^b^	6.12 ± 0.21 ^b^	37.52 ± 4.19 ^b^	4.27 ± 1.06 ^b^	7.51 ± 2.22 ^a^	21.98 ± 1.39 ^a^	14.27 ± 2.13 ^a^	3.41 ± 1.26 ^a^
TT Group	Cocks	Breast	6.17 ± 0.29	6.04 ± 0.30	38.33 ± 4.41 ^B^	3.08 ± 1.34 ^B^	5.70 ± 1.48 ^C^	24.89 ± 4.45 ^B^	13.71 ± 2.29 ^B^	3.02 ± 1.01 ^C^
		Leg	6.24 ± 0.21 ^B^	6.17 ± 0.22 ^B^	33.94 ± 4.32 ^B^	14.92 ± 2.38 ^B^	4.73 ± 2.12 ^C^	26.42 ± 3.89 ^B^	18.98 ± 3.65 ^B^	3.00 ± 1.21 ^A^
	Hens	Breast	6.20 ± 0.29 ^b^	6.17 ± 0.27	39.96 ± 3.38 ^b^	4.01 ± 1.68 ^b^	5.91 ± 2.99 ^c^	31.97 ± 3.67 ^c^	18.61 ± 2.16 ^c^	1.84 ± 0.57 ^c^
		Leg	6.30 ± 0.18 ^a^	6.16 ± 0.19 ^b^	37.38 ± 7.43 ^b^	4.69 ± 0.89 ^c^	3.60 ± 1.08 ^b^	31.99 ± 5.90 ^b^	21.63 ± 3.15 ^b^	1.79 ± 0.96 ^b^

The superscripts ^A–C^ and ^a–c^ were used to separate means within a column for significant differences (*p* < 0.05) in the cocks and hens groups, respectively. The means among column without these superscripts indicate a non-significant difference between groups (*p* > 0.05).

## Data Availability

The data supporting the findings of this study are available on request from the corresponding authors.
